# ^99m^Technetium Sestamibi-^123^Iodine Scintigraphy Is More Accurate Than ^99m^Technetium Sestamibi Alone before Surgery for Primary Hyperparathyroidism

**DOI:** 10.1155/2015/391625

**Published:** 2015-02-01

**Authors:** Eeva M. Ryhänen, Jukka Schildt, Ilkka Heiskanen, Mika Väisänen, Aapo Ahonen, Eliisa Löyttyniemi, Camilla Schalin-Jäntti, Matti J. Välimäki

**Affiliations:** ^1^Division of Endocrinology, Department of Medicine, Helsinki University Central Hospital, University of Helsinki, 00029 Helsinki, Finland; ^2^HUS Medical Imaging Center, Department of Clinical Physiology and Nuclear Medicine, Helsinki University Central Hospital, 00029 Helsinki, Finland; ^3^Department of Surgery, Helsinki University Central Hospital, 00029 Helsinki, Finland; ^4^Department of Biostatistics, University of Turku, 20520 Turku, Finland

## Abstract

*Objectives*. Studies comparing outcome of single-^99m^Tc-methoxyisobutylisonitrile (^99m^Tc-sestamibi) and dual-tracer ^99m^Tc-sestamibi scintigraphy in combination with ^123^I before primary surgery of primary hyperparathyroidism (PHPT) are scarce. *Methods*. We compared ^99m^Tc-sestamibi/^123^I and ^99m^Tc-sestamibi in a single-centre retrospective series of 269 PHPT patients. The results were related to laboratory, surgical and histological findings. *Results*. ^99m^Tc-sestamibi/^123^I and ^99m^Tc-sestamibi were positive in 206 (76.6%) and 111 (41.3%) of 269 patients, respectively (*P* < 0.001). Accuracies for ^99m^Tc-sestamibi/^123^I and ^99m^Tc-sestamibi were 63.4% and 34.9%, respectively (96% CI, *P* < 0.001). Prevalence of multiglandular disease was 15.2%. In multiglandular disease, ^99m^Tc-sestamibi/^123^I and ^99m^Tc-sestamibi revealed 43.8 and 22.1% of pathological glands, respectively (*P* < 0.001). Cure rate was similar for patients with (191/206; 92.7%) and without (59 of 63; 93.7%) a positive ^99m^Tc-sestamibi/^123^I finding. Duration of targeted surgery (one or two quadrants) was 21 and 15 minutes shorter than bilateral neck exploration, respectively (both *P* < 0.001). Higher serum calcium (*P* = 0.014) and PTH (*P* = 0.055) concentrations and larger tumours (*P* < 0.001) characterized the 206 patients with a positive preoperative scan who were cured by removal of a single adenoma. *Conclusions*. ^99m^Tc-sestamibi/^123^I scintigraphy is more accurate than ^99m^Tc-sestamibi before surgery of PHPT. However, outcome of surgery is not determined by scintigraphy alone.

## 1. Introduction

Bilateral neck exploration (BNE) with identification of all four parathyroid glands and with removal of the abnormal gland(s) has been the surgical gold standard for treatment of primary hyperparathyroidism (PHPT). In experienced hands, it is successful in more than 95% of cases without any preoperative imaging [1]. However, as 80 to 85% of patients with PHPT have single-gland disease, targeted surgery such as unilateral neck exploration (UNE) or minimally invasive parathyroidectomy (MIP) may be considered. Focal approaches are favoured by shorter operation times and reduced risk of complications, the major one being recurrent laryngeal nerve palsy. Preoperative imaging studies are mandatory if the surgeon opts for focal procedures. The two main reasons for failed surgery are ectopic glands and undetected multiglandular disease, which is present in about 15% of PHPT patients [2]. Therefore, a major challenge in order to avoid inadequate surgery is accurate preoperative imaging of multiglandular disease [2].

Ideally, preoperative imaging should accurately identify on which side of the neck the pathological parathyroid gland(s) reside, without false positive findings on the healthy side. As introduced by Coakley et al. in 1989 [3], ^99m^Tc methoxyisobutylisonitrile (^99m^Tc-sestamibi) scintigraphy has since gained great popularity and is worldwide probably the most common imaging technique used before primary surgery of PHPT. ^99m^Tc-sestamibi is used either alone or in combination with ^123^iodine (^123^I) or ^99m^technetium pertechnetate (^99m^TcO_4_^−^) for subtraction of the thyroid image, as uptake of ^99m^Tc-sestamibi is not tissue specific. ^99m^Tc is sequestered within mitochondria in both thyroid and parathyroid glands, salivary tissue, and normal cardiac cells [2, 4, 5]. Imaging with the single-tracer technique is based on the different washout kinetics for thyroid and parathyroid tissue. Maximum thyroid gland activity is reached within five minutes while parathyroid activity is sustained with delayed washout, allowing for acquisition of the parathyroid glands two hours after injection [6]. Thyroid nodules may have a prolonged washout time and cause false-positive findings in single-tracer scintigraphy with ^99m^Tc-sestamibi [2]. The dual-tracer technique is performed in combination with^ 123^I or ^99m^Tc, which are taken up by thyroid tissue only. The ^123^I or ^99m^Tc thyroid image is thereafter digitally subtracted from the ^99m^Tc-sestamibi image, allowing for visualization of parathyroid tissue alone [6]. Some studies compared the sensitivity and specificity of neck ultrasound and ^99m^Tc-sestamibi scintigraphy for revealing hyperfunctioning parathyroid glands, demonstrating similar performance [7]. A preoperative study in 15 PHPT patients comparing the performance of ^99m^Tc-sestamibi SPECT with ^99m^Tc-sestamibi/^123^I SPECT suggested that the double-tracer technique performs better [6] and a series of 37 PHPT patients also concluded that imaging with the dual-tracer protocol is more accurate than imaging with a single tracer [8]. Despite being widely used, single centre studies comparing the performance of ^99m^Tc-sestamibi/^123^I to that of ^99m^Tc-sestamibi alone in large patients series referred for surgery of primary PHPT are scarce.

At our centre, routine preoperative ^99m^Tc-sestamibi scintigraphy has been performed for more than 15 years. We used the single-tracer technique up to November 2006, whereafter the double-tracer ^99m^Tc-sestamibi/^123^I technique was introduced. The aim of the present study was to retrospectively compare the performance of these two techniques in 269 consecutive patients who underwent primary surgery at our centre during years 2006–2009 because of primary PHPT.

## 2. Patients and Methods

### 2.1. Patients

**Figure 1:**
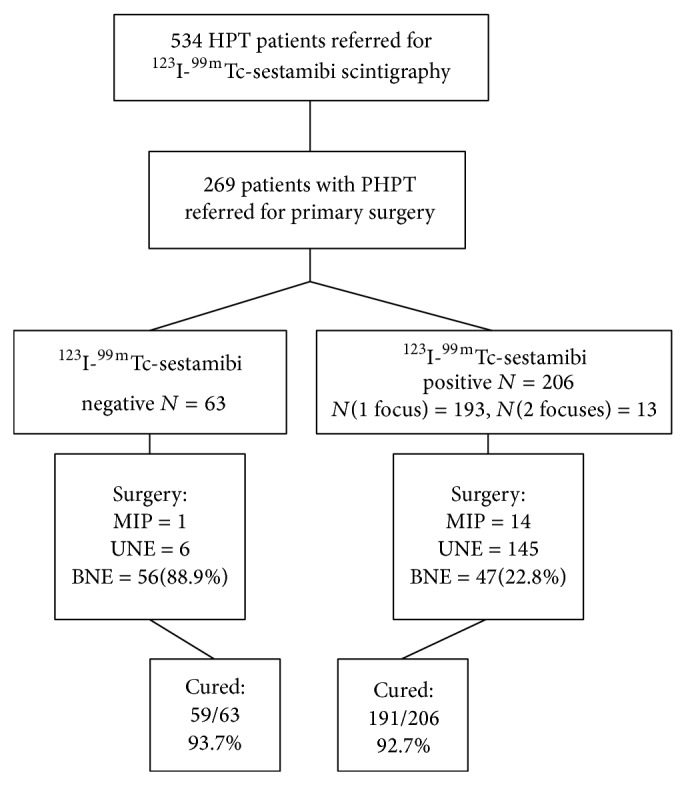
Flow chart of 534 patients referred for ^99m^Tc-sestamibi/^123^I scintigraphy because of hyperparathyroidism (HPT). MIP: mini-invasive parathyroidectomy; UNE: unilateral exploration; BNE: bilateral exploration.

From November 2006 through November 2009, 543 consecutive patients were referred for evaluation of surgery because of suspected PHPT to the Helsinki University Hospital. All patients underwent parathyroid scintigraphy at the Helsinki University Hospital Medical Imaging Center, Department of Clinical Physiology and Nuclear Medicine. From this original cohort we excluded patients suffering from secondary or tertiary hyperparathyroidism and patients who were not operated on (Figure 1). Further exclusion criteria were patients referred for reoperation of PHPT and patients suffering from MEN-1 syndrome. The final patient series comprised 269 subjects who all fulfilled the biochemical criteria for PHPT and also fulfilled the criteria for operative treatment of PHPT [9].

The multiglandular disease (MGD) group was defined as the patients who were not cured by removal of one abnormal parathyroid gland and patients from whom two or more pathological glands were surgically removed. The number of pathological glands in uncured MGD patients was estimated by adding one gland to the total number of abnormal glands removed. Short-term remission (normal serum calcium and at least 50% decrease in serum PTH concentrations) was evaluated. The study was conducted according to the guidelines of the Declaration of Helsinki. The protocol was approved by an institutional board review.

### 2.2. Scintigraphy

Patients fasted for two hours before the study. A ^123^I-iodide capsule of 13–15 MBq was administered orally 3 hours before the ^123^I-imaging. Immediately thereafter, 790–940 MBq of ^99m^Tc-MIBI was intravenously injected and data acquisition started in 5 minutes. A double-window procedure was used to acquire simultaneous imaging of both radiopharmaceuticals. Late imaging with a similar double-window procedure was performed two hours after the first imaging. A subtraction procedure, in which ^123^I images were subtracted from ^99m^Tc-MIBI images by means of manually drawn regions of interest (ROI), was performed for both early and late imaging. Dual-phase MIBI technique comprised early and late ^99m^Tc-MIBI images.

The imaging was performed with a triple-head Picker Prism 3000XP gamma camera (Cleveland, OH, USA) equipped with a Picker Odyssey FX Computer. A low energy general all-purpose collimator was used for imaging. The image matrix was 256 × 256. Window settings were 140 keV ± 8% for ^99m^Tc and 159 keV ± 7% for ^123^I.

A focally increased activity in ^99m^Tc-MIBI images and an abnormal washout and residual uptake on subtraction images were interpreted as a positive finding. The location of the abnormal uptake was reported. To confirm the quality of original scintigraphy findings, all images were blindly reevaluated by two experienced nuclear medicine physicians (Jukka Schildt and Aapo Ahonen). If their opinions differed, they evaluated the images together to reach a consensus. The findings were divided into four different categories: normal, equivocal, slightly suspicious, and abnormal. For data analyses, equivocal findings were considered negative. In 13 patients (4.8%) the reevaluation differed from the original ^99m^Tc-sestamibi/^123^I scintigraphy reviews, which guided the operation and were used in the analyses. The MIBI alone reviews were obtained from the reevaluation of the images.

Per-patient based scintigraphy was considered accurate if the lesion(s) detected by the localization technique were surgically removed and were histologically confirmed to be adenoma, hyperplasia, or carcinoma, and the patient was biochemically cured. Findings that were not verified on surgery or histologically or findings that did not result in biochemical cure after operation of the corresponding site were considered not accurate. As all patients had biochemical evidence of the disease, negative preoperative localization studies were considered false negative, that is, not accurate.

### 2.3. Surgery

The type of surgery was chosen on the basis of the ^99m^Tc-sestamibi/^123^I review available at the time of surgery and clinical data. Any condition indicating hyperplasia favoured bilateral neck exploration (BNE). BNE was performed through Kocher's suprajugular incision, with an attempt to identify all four parathyroid glands and remove obviously enlarged and pathological ones. In the unilateral scan-directed approach (UNE), only the side of the expected pathological parathyroid gland was dissected [10]. Focused parathyroidectomy was performed either through standard Kocher's suprajugular incision or a minimal skin incision (<25 mm) with excision of a solitary parathyroid tumor; further dissection to identify another ipsilateral parathyroid gland was not performed. In open minimal invasive parathyroidectomy (MIP) an incision was made anterolaterally close to the site of the suspected parathyroid adenoma. If an abnormal parathyroid gland was not found in the scan-positive location, another ipsilateral gland was exposed and removed if clearly enlarged. When necessary, a focused minimal invasive procedure was converted to BNE. In equivocal cases, a frozen sample was examined by a pathologist before completing the operation. Intraoperative PTH measurements were not used.

### 2.4. Statistics

For biochemically cured patients the comparisons between those with a single adenoma detected or not detected on preoperative ^99m^Tc-sestamibi/^123^I scan were performed using the Wilcoxon rank sum test or the chi-square test. McNemar's and Fisher's exact tests were used to compare operative and imaging findings and the accuracy between ^99m^Tc-sestamibi/^123^I and ^99m^Tc-sestamibi scans. The duration of the operative procedures was log-transformed before comparison according to the type of surgery with one-way analysis of variance. Pairwise comparisons between the different types of surgery were performed with Tukey's method. A *P* value less than 0.05 was considered statistically significant. All tests were performed as two-sided tests. S+ Software, version 8.1, was used for statistical analyses.

## 3. Results

A flow chart of the study is depicted in Figure 1. Baseline characteristics of the 269 study subjects are given in Table 1. ^99m^Tc-sestamibi/^123^I revealed one (*n* = 193) or two (*n* = 13) positive findings in 206 patients (76.6%). ^99m^Tc-sestamibi alone revealed significantly fewer focuses, one (*n* = 102) and two (*n* = 9) in 111 patients (41.3%; *P* < 0.001). In two patients lesions were detected by ^99m^Tc-sestamibi alone. Unilateral operation was performed in 166 patients; 97 operations were targeted to one quadrant, 54 operations were targeted to two quadrants, and 15 operations were minimally invasive parathyroidectomy. One hundred and three patients underwent bilateral neck exploration (38.2% of study patients). Bilateral neck exploration was performed in 88.9% of patients with negative preoperative imaging and in 22.8% with positive preoperative imaging results.

### 3.1. Outcome of Surgery

Two hundred and forty-nine patients (92.5%) were cured by removal of pathological parathyroid glands. In addition, one patient was biochemically cured after removal of a retrosternal goiter, although the abnormal parathyroid gland(s) was not identified at surgery. The cure rate was similar for patients with (191 of 206; 92.7%) and without (59 of 63; 93.7%) a positive finding on ^99m^Tc-sestamibi/^123^I (*P* = NS) (Figure 1). Surgery was not successful in 19 patients. Sixteen failures (84.2%) were related to multiglandular disease and in three cases no abnormal parathyroid gland was found. Twelve, three, and four of the 19 patients had unilateral, bilateral, or no finding on ^99m^Tc-sestamibi/^123^I scintigraphy, respectively.

### 3.2. Comparison of Scintigraphic and Surgical Findings

#### 3.2.1. Accuracy

Overall accuracies of the whole study population (95% CI) for ^99m^Tc-sestamibi/^123^I and ^99m^Tc-sestamibi were 63.4% (57.4–69.0) and 34.9% (29.2–40.6), respectively (*P* < 0.001) (Table 2). For the whole cohort, accuracies for detection of one abnormal parathyroid gland were 60.9% (54.9–66.8) for ^99m^Tc-sestamibi/^123^I and 34.2% (28.5–40.2) for ^99m^Tc-sestamibi, respectively (*P* < 0.001). The corresponding accuracies for MGD (= the scan correctly revealed all pathological glands) were 14.6% (3.8–25.5) and 9.7% (0.6–18.8), respectively (*P* < 0.001).

#### 3.2.2. Unilateral Uptake on ^99m^Tc-Sestamibi/^123^I Scintigraphy

**Figure 2:**
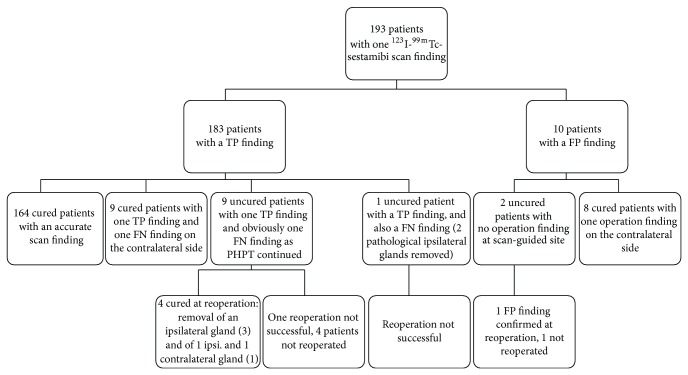
The findings and outcome of surgery in 193 patients with a unilateral uptake on ^99m^Tc-sestamibi/^123^I scintigraphy. TP: true positive scintigraphy finding; that is, a pathological parathyroid gland was found at the scan-guided site at surgery. FP: false positive scintigraphy finding; that is, no pathological gland was found at the scan-guided site at surgery. FN: false negative scintigraphy finding; that is, a pathological gland was identified at surgery despite no positive preoperative imaging finding at this site, or a patient was not cured despite the removal of a gland identified by preoperative imaging.

**Figure 3:**
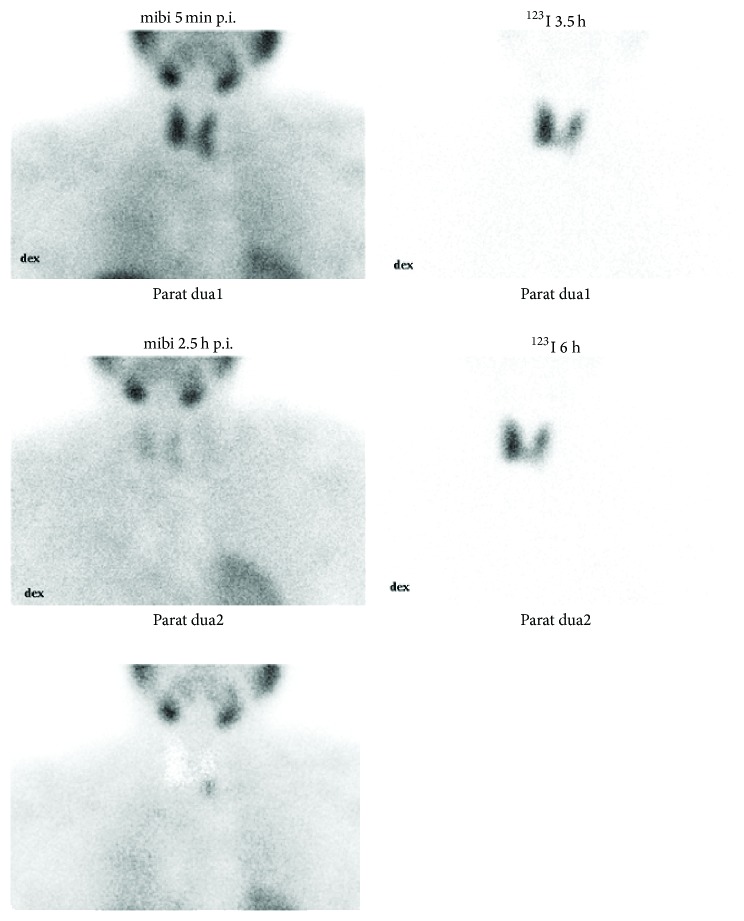
Parathyroid ^99m^Tc-sestamibi-^123^I-subtraction scintigraphy in a 53-year-old woman with primary hyperparathyroidism (serum ionized calcium 1.58 mmol/l, PTH 201 ng/l). The patient was cured by removal of a 2.0-gram parathyroid adenoma excised at the site predicted in the scintigraphy. First row: early ^99m^Tc-sestamibi image (left), early ^123^I image (right). Second row: delayed ^99m^Tc-sestamibi image (left), delayed ^123^I image (right). Third row: the subtraction image of delayed ^99m^Tc-sestamibi and ^123^I images shows a focal uptake at the inferior pole of left thyroid gland and is seen only in this image.

Among 193 patients (71.7%) with one focus on ^99m^Tc-sestamibi/^123^I, the surgeon identified one pathological gland at the scan-guided site in 183 patients, but only 164 of these patients were cured after surgery because of additional diseased parathyroid glands (Figure 2). In 10 patients with a unilateral ^99m^Tc-sestamibi/^123^I finding, surgery was extended but did not result in cure (Figure 2). In 19 of 193 (9.8%) patients there was a true positive finding but also a false negative finding. Ten patients (5.2% of 193 cases) had a single false positive uptake on ^99m^Tc-sestamibi/^123^I (Figure 2). In altogether 29 of 193 patients (15%) ^99m^Tc-sestamibi/^123^I failed to accurately identify all pathological glands (Figure 2). Twenty-two of these 29 patients were cured either by extended primary surgery or reoperation. Other inaccuracies included 10 patients with bilateral disease for whom ^99m^Tc-sestamibi/^123^I demonstrated only one unilateral uptake and 9 patients with a single uptake on the wrong side of the neck. In 3 cases primary surgery was unsuccessful despite a correct finding on ^99m^Tc-sestamibi/^123^I (confirmed at reoperation). In 7 cases disease status remains open as reoperation was unsuccessful or not performed (Figure 2).  Figure 3 demonstrates typical findings in a patients for whom dual isotope scintigraphy was positive and single isotope scintigraphy remained negative.

#### 3.2.3. Bilateral Uptake on Preoperative ^99m^Tc-Sestamibi/^123^I Scintigraphy

Thirteen patients (4.8%) had bilateral uptake on ^99m^Tc-sestamibi/^123^I and 10 (76.9%) were cured. Eleven underwent bilateral and two unilateral neck exploration. Based on the operative findings at primary surgery, ^99m^Tc-sestamibi/^123^I was considered accurate in 6/13 (46.2%), but the number rose to 9/13 (69.2%) based on reoperative findings. Four of 13 patients who had single-gland disease but a false positive finding on the other side of the neck were cured.

#### 3.2.4. Negative Preoperative ^99m^Tc-Sestamibi/^123^I

^99m^Tc-sestamibi/^123^I demonstrated no uptake in 63 patients (23.4%), 56 of whom underwent bilateral exploration (Figure 1). Surgery revealed one pathological gland in 57 of the 63 patients, 54 of whom were biochemically cured. Four of the 63 patients proved to have multiglandular disease and were cured after bilateral exploration. Although no apparent parathyroid tissue was removed in two of the 63 patients, one of them was biochemically cured.

### 3.3. Comparison of Scintigraphic and Histopathological Findings

Histopathology confirmed single adenoma in 218 patients. ^99m^Tc-sestamibi/^123^I was accurate in 75.7% and ^99m^Tc-sestamibi alone in 41.7% (*P* < 0.001) of cases (Table 3). For the 47 patients with other histological diagnoses or two or more abnormal glands (multiple adenomas, hyperplasia, a combination of these, or carcinoma and hyperplasia), 37/76 (48.7%) pathological glands were accurately revealed by ^99m^Tc-sestamibi/^123^I. Five patients had parathyroid carcinoma (3 had carcinoma only and 2 had both parathyroid carcinoma and hyperplasia). All parathyroid carcinomas were visible on ^99m^Tc-sestamibi/^123^I.

### 3.4. Multiglandular Disease

Sixteen patients were not cured by removal of one abnormal parathyroid (12 adenomas, 4 hyperplastic glands) and were thus considered as having multiglandular disease. In addition, two or more abnormal glands were removed at primary surgery in 25 patients with altogether 58 abnormal parathyroids. The rate of multiglandular disease in the whole cohort was 15.2% (41/269 patients) and per-patient based cure rate was 61.0% (25/41 patients).

### 3.5. Determinants of True Positive ^99m^Tc-Sestamibi/^123^I Findings

Two hundred and six patients were cured by removal of a single adenoma. Preoperative serum ionized calcium and PTH concentrations were significantly higher in the 159 patients whose preoperative scan demonstrated one focus compared to the 47 patients who had a negative scan (*P* < 0.02 and *P* < 0.01, resp.; Table 4). Median weight of the removed adenomas was twice higher in scan-positive compared to scan-negative patients (*P* < 0.001; Table 4).

### 3.6. Duration of Surgery

The duration of surgery according to the type of surgery performed is given in Table 5. Median operation time for surgery targeted to one or two quadrants was 21 and 15 minutes shorter compared to bilateral neck exploration, respectively (both *P* < 0.001). Targeted one-quadrant surgery was 6 minutes shorter than unilateral neck exploration (*P* < 0.001).

## 4. Discussion

The main finding of this single-centre retrospective series of 269 consecutive patients is that ^99m^Tc-sestamibi/^123^I scintigraphy is significantly more accurate than ^99m^Tc-sestamibi scintigraphy alone (63% versus 34%) in the workup of PHPT patients referred for primary surgery. The findings are in line with some previous studies reporting better performance of dual- compared to single-tracer techniques [8, 10–12]. However, these studies reported sensitivities (72%–94% versus 62%–79%) rather than accuracies [8, 10–12]. A recent Finnish study comparing 5 different scintigraphy protocols in 24 patients concluded that any dual-tracer protocol with ^99m^Tc-sestamibi and ^123^I is superior to ^99m^Tc-sestamibi alone [13]. Philippon et al. compared the preoperative findings of ^99m^Tc-sestamibi/^123^I and ^99m^Tc-sestamibi alone in 182 patients who underwent bilateral neck exploration because of PHPT and reported accuracies of 50% and 32%, respectively [14]. Wakamatsu et al. [15] reported a sensitivity of 39% for ^99m^Tc-sestamibi alone. The findings of the present study are in line with the recommendations of the EANM guidelines; that is, an additional thyroid-specific isotope should be used in order to get a pure thyroid image [2]. SPECT or SPECT/CT has gained much popularity for localization of pathological parathyroid glands, and CT allows for better anatomical identification of the abnormal gland. However, SPECT or SPECT/CT was not used in the present study. However, we previously reported (Schalin-Jäntti et al.) [16] that, in PHPT, planar ^99m^Tc-sestamibi/^123^I performs better than ^99m^Tc-sestamibi SPECT before reoperation. This is in line with the study by Tunninen et al. [13] who found no difference in sensitivity, specificity, or accuracy between the acquisition techniques using dual-tracer technique (planar, SPECT, or SPECT/CT imaging). The data indicate that using dual isotopes instead of single isotope is more crucial than planar, SPECT, or SPECT/CT imaging per se. Hassler et al. [17] studied SPECT/CT and planar imaging with ^99m^Tc-sestamibi/^123^I and reported no significant difference in sensitivities (86% and 75%, resp.) but significant difference in specificities (100% and 90%, resp.).

In the present study, ^99m^Tc-sestamibi remained negative in up to one-quarter of the patients. In line with previous results, patients with negative preoperative scintigraphy were characterized by mild single-gland disease, mild increases in serum calcium and PTH concentrations, and only slightly enlarged pathological parathyroid glands [14, 15, 18–21]. Small tumour size and mild disease are therefore important negative determinants of the preoperative scan.

It has been estimated that approximately 60–70% of patients with PHPT are candidates for unilateral neck exploration [15, 19–21]. In order to be of help, preoperative imaging must unequivocally demonstrate single-gland disease in patients scheduled for targeted surgery. Furthermore, patients with familial PHPT or multiple endocrine neoplasia must be excluded, as well as patients who have undergone previous parathyroid or thyroid surgery. In the present series, 193 (71.5%) patients fulfilled these criteria. Targeted surgery failed in 6% (12/166) of patients undergoing unilateral neck exploration or a minimal invasive approach. If targeted surgery had not been converted to bilateral neck exploration, 17 additional cases would have been unsuccessful. When taking into account the results of later reoperations, overall false positive and false negative rates of the 206 positive preoperative ^99m^Tc-sestamibi/^123^I scans in the present study were 6.8% (14/206) and 9.2% (19/206), respectively. In comparison, in the large study by Civelek et al., using delayed sestamibi-SPECT imaging only, a higher false positive rate of 14% (58/407) was reported [20].

In the present series, 16/19 patients with unsuccessful surgery had multiglandular disease, and, in line with the study by Hindié et al. [22], the rate of multiglandular PHPT was at least 15%. Siperstein et al. [23] reported an even higher percentage of 22 in a series of 1158 patients with a single finding on sestamibi imaging, all of whom underwent bilateral neck exploration in addition to targeted surgery. Compared to the ^99m^Tc-sestamibi single-tracer technique [18], higher sensitivities for subtraction imaging have been reported also for multiglandular disease, exceeding 80% in a study by Hindié et al. [22]. In the present series, 43.8% of the total number of positive ^99m^Tc-sestamibi/^123^I findings in multiglandular disease were true positive. Thus, a serious limitation of preoperative imaging for PHPT is failure to accurately identify multiglandular disease. Shen et al. concluded that their surgical failure rate would be as high as 10% if surgery was based on sestamibi scan imaging instead of routine bilateral neck exploration [24]. Furthermore, Norman et al. reported recurrent disease in at least 5% during 10-year follow-up of patients initially held as cured by primary unilateral surgery [25]. In contrast, the results of primary bilateral neck exploration did not change over time [25].

In accordance with previous studies [26], ^99m^Tc-sestamibi/^123^I scintigraphy did not improve outcome of surgery in the present series. However, a single positive finding aids the surgeon in selecting the correct patients for targeted surgery. Limited exploration enables shorter operation time [26–28] and is generally associated with lower complication rates. Most surgeons appreciate having preoperative information regarding which side of the neck dissection should be initiated. The third international workshop on surgery for PHPT concluded that the main advantage of sestamibi scans is the ability to localize parathyroid glands in ectopic sites, including the mediastinum [29]. It was also concluded that surgery should not be ruled out based on a negative preoperative scan [29]. Although most previous localization studies relate operative success primarily to the sensitivity of the preoperative imaging technique, choice of surgery is another important determinant. In the present study, the reading of the scan changed in 5% upon reevaluation. Great care should thus also be given to adequate reading of the scans.

In conclusion, this large retrospective series demonstrates that ^99m^Tc-sestamibi/^123^I is more accurate than ^99m^Tc-sestamibi alone in the preoperative workup of PHPT. However, performing scintigraphy is not equal to improving surgical outcome. Not even ^99m^Tc-sestamibi/^123^I performs well in multiglandular disease, which is present in about 15% of patients referred for primary surgery of PHPT.

## Figures and Tables

**Table 1:** Characteristics of 269 patients with primary hyperparathyroidism (median with range or *n* with percentage).

	Median (range) or *n* with percentage
Age (years)	61 (16–91)

Gender: female/male	199 (74%)/70 (26%)

Serum ionized calcium (mmol/L)	1.49 (1.30–2.42)

Serum PTH (ng/L)	159 (53–3765)

Serum 25-OHD (nmol/L) (*n* = 224)	42.5 (13–99)

Serum creatinine (*μ*mol/L)	68 (42–330)

Urinary calcium (mmol/24 h) (*n* = 252)	8.76 (0.23–29.38)

Number of focuses on ^123^I-^99m^Tc-sestamibi scan (0/1/2%)	63/193/13 (23,4/71,7/4,8)

Number of focuses on ^123^I-^99m^Tc-sestamibi scan (0/1/2%)	158/102/9 (58,7/37,9/3,3)

Weight of pathological gland (g) (*n* = 188)	0.65 (0.037–22)

**Table 2:** Accuracies of preoperative localization studies.

	Planar scintigraphy with ^99m^Tc-sestamibi	Planar scintigraphy with ^123^I-^99m^Tc-sestamibi
Overall accuracy (95% CI)	34.9% (29.2–40.6)	63.4% (57.4–69.0)
Accuracy for one scintigraphic finding	34.2% (28.5–40.2)	60.9% (54.9–66.8)
Accuracy in multiglandular disease^*^	9.7% (0.6–18.8)	14.6% (3.8–25.5)

*P* value < 0.001 between ^123^I-^99m^Tc-sestamibi and ^99m^Tc-sestamibi-scintigraphy.

^*^The scan revealed correctly all pathological glands without false negative findings.

**Table 3:** Scintigraphic results compared with histopathological findings in 265 patients who had pathological gland(s) removed.

Histopathology	Patients *n*	Cured *n* (%)	Number of pathological glands	Number (%) of positive glands on ^123^I-^99m^Tc-sestamibi scan	True positives^a^ *n* (% of all glands)	False positive^b^ (*n*)
Single adenoma	218	206 (94)	218	176 (81)	165 (76)	11^c^
Multiple adenomas	3	3	6	3 (50)	3 (50)	0
Hyperplasia (one gland in 10^d^ cases)	24	20	40	21 (52)	18 (45)	3
Hyperplasia or/and adenoma (one gland in 9 cases)	15	15	22	12 (54)	11 (50)	1
Carcinoma	3	3	3	2 (67)	2 (67)	0
Carcinoma and hyperplastic gland	2	2	5	3 (60)	3 (60)	0

^a^A true positive finding is defined as scintigraphic finding on the same side as the operative finding, and the finding is histologically confirmed.

^b^A false positive finding is defined as scintigraphic finding on the contralateral side compared to the operative finding.

^c^Of these 11 patients, 7 had bilateral imaging findings and 4 had one unilateral finding on the wrong side of the neck.

^d^Of the 10 patients with one hyperplastic gland removed at primary surgery, 4 were not cured as they had multiglandular disease, while 4 had single gland disease and were cured.

**Table 4:** Characteristics (median with range) of cured patients with a single adenoma (*n* = 206) detected or not detected on preoperative ^123^I-^99m^Tc-sestamibi scintigraphy.

	Detected (*n* = 159)	Not detected (*n* = 47)	*P* value
Age (years)	61 (31–91)	63 (30–81)	0.57
Gender: female/male	116 (73%)/43	33 (58%)/14	0.71
Serum ionized calcium (mmol/L)	1.46 (1.3–2.24)	1.43 (1.3–1.74)	0.014
Serum PTH (g/L)	168 (71–1887)	133 (82–388)	0.0055
Serum 25-OHD (nmol/L)	44 (14–98)	48 (22–67)	0.68
Serum creatinine (mmol/L)	69 (45–225)	66 (46–137)	0.43
Urinary calcium (mmol/24 h)	9.02 (0.49–29.38)	8.00 (0.69–18.56)	0.37
Weight of adenoma (g)	0.78 (0.08–8.49)	0.35 (0.13–1.30)	<0.001

**Table 5:** The duration (min; median with range) of operative procedure among 219 cured patients, who did not have simultaneous thyroid surgery.

Type of surgery	*n*	Duration
Unilateral, one quadrant^*^	105	46.2 (22.0–135.0)
Unilateral, two quadrants	44	52.4 (33.0–160.0)
Bilateral neck exploration	70	67.9 (33.0–181.2)

*P* value < 0.001 for comparison between the groups.

^*^Comprising mini-invasive surgery.
